# Integrative bioinformatics analysis identifies placental senescence-associated signatures in early-onset preeclampsia

**DOI:** 10.3389/fendo.2026.1863608

**Published:** 2026-07-08

**Authors:** Li Lin, Ying Chen, Lei Chen, Shengyi Gu, Yao Lai, Xiang Li, Jing Peng, Xiaolin Hua

**Affiliations:** 1 Shanghai Key Laboratory of Maternal Fetal Medicine, Shanghai Institute of Maternal-Fetal Medicine and Gynecologic Oncology, Shanghai First Maternity and Infant Hospital, School of Medicine, Tongji University, Shanghai, China; 2 Department of Obstetrics, The International Peace Maternity and Child Health Hospital, School of Medicine, Shanghai Jiaotong University, Shanghai, China

**Keywords:** early-onset preeclampsia, leptin, machine learning, placental senescence, single-cell RNA sequencing

## Abstract

**Background:**

Early-onset preeclampsia (EOPE) is a severe hypertensive disorder of pregnancy associated with preterm delivery and maternal multi-organ dysfunction. Although placental senescence and immune dysregulation have been implicated in EOPE, the expression profile of senescence-related genes(SRGs) and their mechanistic roles in disease progression remain unclear.

**Methods:**

Multiple placental bulk RNA-seq datasets were integrated to identify senescence-related differentially expressed genes in EOPE. Weighted gene co-expression network analysis (WGCNA) and multiple machine learning algorithms were used to identify hub genes. Single-cell RNA sequencing was further applied to define the cellular expression patterns of hub genes and characterize senescence-associated changes in EOPE. Key findings were subsequently validated in clinical placental specimens using molecular and functional experiments.

**Results:**

A total of 44 senescence-related differentially expressed genes were identified in EOPE placentas and were mainly enriched in cell proliferation-related pathways. LEP, ENG, MIF, and CYBB were identified as hub genes and were predominantly expressed in trophoblasts and immune cells. Single-cell analysis showed that senescence-associated activity was primarily enriched in the trophoblast lineage and further implicated LEP in syncytiotrophoblast (SCT) senescence and SCT secretory dysfunction, suggesting a role in EOPE pathogenesis.

**Conclusions:**

Our findings suggest that EOPE is closely related to placental senescence. Deeper investigation of SRGs and pathways may aid early diagnosis and clarify the molecular basis of EOPE.

## Introduction

Preeclampsia (PE) is a pregnancy-specific hypertensive disorder affecting approximately 5%–8% of all pregnancies worldwide and remains a major cause of maternal and perinatal morbidity and mortality (1, 2). Clinically, PE manifests as new-onset hypertension occurring after 20 weeks of gestation, along with multisystem disorders including proteinuria, hepatic and renal dysfunction. PE is commonly classified into early-onset preeclampsia (EOPE, <34 weeks of gestation) and late-onset preeclampsia (LOPE, ≥34 weeks of gestation) according to gestational age at clinical onset (3). EOPE is generally associated with more severe placental dysfunction, greater fetal growth restriction, and poorer perinatal outcomes than LOPE (4). Therefore, clarifying its pathogenesis and developing effective interventions are of major clinical importance. Notably, placental delivery remains the only definitive treatment for PE (5), highlighting the pivotal role of the placenta in disease pathogenesis.

Aging is increasingly recognized not only as a physiological process but also as a fundamental driver of diverse pathological conditions, characterized by cellular senescence, genomic instability, and loss of tissue homeostasis (6, 7). Premature placental aging may impair placental function and has been implicated in placenta-mediated disorders, including PE (8–10). Consistent with this, a population-based serum proteomic study identified enrichment of placental aging-related pathways in maternal cardiac dysfunction and heart failure during pregnancy (11). Furthermore, Nonn et al. using single-nucleus RNA sequencing, showed that multinucleated syncytiotrophoblasts(SCT) exhibit aberrant regulation as early as the first trimester, together with increased expression and secretion of senescence-associated secretory phenotype (SASP) factors, including activin A and GDF15 (12). These findings support a potential role for placental senescence, particularly exaggerated SCT senescence and dysfunction, in the pathogenesis of EOPE.

Although senescence has increasingly been implicated in the pathogenesis of PE, the global landscape and potential functions of senescence-related genes in EOPE remain poorly defined. In particular, placental cells exhibit progressively increased senescence signatures with advancing gestational age, raising the possibility that physiological placental aging (13, 14) may confound the identification of disease-associated premature senescence. To distinguish EOPE-associated pathological senescence from normal gestational maturation, we integrated multiple placental transcriptomic datasets comprising EOPE and gestational age-matched preterm birth (PTB) controls and combined bulk transcriptomic, machine-learning, single-cell RNA sequencing, and trajectory analyses. The aim of this study was to systematically characterize senescence-related molecular alterations in EOPE, identify key senescence-associated genes, and explore their potential roles in placental dysfunction and disease pathogenesis.

## Materials and methods

### Dataset source, preprocessing and analysis

We conducted an integrative transcriptomic analysis to elucidate senescence-associated mechanisms in EOPE. Publicly available placental bulk RNA-seq datasets, including GSE114691 (15), GSE203507 (16), and GSE74341 (17), were retrieved from the Gene Expression Omnibus (GEO) database (https://www.ncbi.nlm.nih.gov/geo/) and collectively served as the discovery cohort. From these datasets, we carefully selected placental samples from EOPE patients and gestational age-matched PTB controls, resulting in a cohort comprising 35 EOPE and 31 PTB cases. An independent dataset GSE44711 (18), was used as an external validation cohort (bulk RNA-seq datasets details in **Supplementary Table S1**).

Data preprocessing and analysis were performed using R software (v4.5.0). For RNA-seq datasets with count-level supplementary files, transcript identifiers were annotated to gene symbols using biomaRt and converted into gene-level expression matrices. For microarray datasets, probe identifiers were mapped to gene symbols using the corresponding platform annotation files. When multiple transcripts or probes mapped to the same gene, the median expression value was retained. Expression matrices were log2-transformed when necessary and normalized using the normalizeBetweenArrays function in the limma package (19). Batch effects among datasets were corrected using the ComBat algorithm implemented in the sva package (20). The effectiveness of normalization and batch correction was evaluated using boxplots and principal component analysis (PCA). Differential expression analysis was subsequently performed using the limma package. Differentially expressed genes (DEGs) were defined as genes with FDR-adjusted P < 0.05 and |log_2_FC| > 0.585 (1.5-fold expression change). Results were visualized with ggplot2.

### Functional enrichment and pathway analysis

To investigate the biological implications of transcriptional dysregulation in EOPE, functional enrichment analyses were conducted using the clusterProfiler R package (21). Prior to enrichment analysis, DEGs were annotated and converted into Entrez Gene IDs based on the org.Hs.eg.db database. Gene Ontology (GO) enrichment analysis, including the categories of biological process (BP), cellular component (CC), and molecular function (MF), was then performed to characterize the functional features of EOPE-specific DEGs. In addition, Kyoto Encyclopedia of Genes and Genomes (KEGG) pathway enrichment analysis was applied to identify significantly perturbed signaling cascades and biological pathways.

### Collection of senescence-related genes of EOPE (SRGs)

Aging-related genes were systematically curated from multiple authoritative aging resources. Senescence-associated genes were obtained from the Human Ageing Genomic Resources (HAGR; https://genomics.senescence.info/) (22), including the core cellular senescence gene set CellAge and the curated human aging gene collection GenAge. In addition, aging-associated genes were extracted from AgingAtlas (23), an independent multi-omics database that provides comprehensive annotations of aging-related genomic features. After merging these datasets and removing duplicate entries, a total of 1,061 SRGs were identified and used as the candidate gene set for subsequent screening and functional analyses.

### WGCNA and screening for SRDEGs with highly correlated EOPE features

Weighted Gene Co-expression Network Analysis (WGCNA) is an algorithm that identifies biologically meaningful co-expressed gene modules to investigate the associations between gene expression patterns and diseases (24). We used the WGCNA R package to identify key co-expressed gene modules linked to EOPE. Sample quality control was conducted via hierarchical clustering, and samples with excessive missing values were removed. The optimal soft-thresholding power (β) was determined to construct a scale-free network.Topological overlap matrix (TOM) construction was conducted based on the gene expression data. Dynamic tree cutting algorithm was adopted to identify distinct co-expression gene modules, and modules with high similarity were further merged subsequently. Module membership (MM) and gene significance (GS) values were computed to assess the correlation between each module and clinical traits. The key module exhibiting the strongest association with EOPE phenotype was screened out for subsequent downstream investigations. To identify senescence-related differentially expressed genes (SRDEGs) strongly correlated with EOPE, we performed intersection analysis of DEGs, WGCNA-derived hub genes, and SRGs. The overlapping genes were visualized using Venn diagrams.

### Analysis of protein–protein interactions (PPI)

PPI networks were performed based on SRDEGs using the STRING database (https://string-db.org/). Hub SRDEGs were identified using the CytoHubba and MCODE plugins in Cytoscape software (version 3.10.0). For MCODE analysis, the filtering thresholds were set as follows: Degree Cutoff = 2, Max Depth = 100, K-Core = 2, and Node Score Cutoff = 0.2. Subsequently, the CytoHubba plugin was utilized to identify hub genes under the criterion that the Matthews correlation coefficient value was no less than 0.6. Finally, the top 10 hub genes were selected by integrating the results from both plugins.

### Machine learning

For the identification of potential candidate genes, the VennDiagram package was used to visualize the overlapping key genes derived from SRDEGs. Four distinct machine learning algorithms were subsequently employed to screen for critical genes closely associated with EOPE. Specifically, least absolute shrinkage and selection operator (LASSO) regression analysis (25) was conducted using the “glmnet” package, while support vector machine–recursive feature elimination (SVM-RFE) (26) analysis utilized the “e1071”, “kernlab” and “caret” packages. In addition, random forest (RF) (27) and eXtreme Gradient Boosting (XGBoost) (28) analyses were carried out using the randomForest and xgboost packages, respectively. Ultimately, four hub SRDEGs identified simultaneously by LASSO, RF, SVM-RFE, and XGBoost were regarded as high-confidence candidate genes associated with EOPE and were selected for subsequent analyses and experimental validation.

### Immune infiltration analysis

To characterize the immune microenvironment alterations associated with placental senescence in EOPE, immune cell infiltration was systematically evaluated using CIBERSORT. This analytical approach was applied to quantify the relative abundance and calculate the enrichment scores of distinct immune cell subsets, which was based on pre-established gene signatures specific to immune cell markers. Differences in immune infiltration between EOPE and PTB groups were assessed using the Wilcoxon rank-sum test. Heatmaps were constructed to visualize immune landscape alterations across cohorts.

### Explanation of machine learning models

Based on the selected predictive factors, ten machine learning models were constructed in this study, including ridge least squares (RLS), random forest (RF), decision tree (DTS), support vector machine (SVM), logistic regression, k-nearest neighbor (KNN), eXtreme Gradient Boosting (XGBoost), gradient boosting machine (GBM), neural network, and generalized linear model boosting (GlmBoost). We evaluated the predictive performance of ten models using the receiver operating characteristic (ROC) curve as the primary metric for disease discrimination. The model with the best predictive performance was selected as the final model for this study. To further enhance the interpretability of the final prediction model, we applied Shapley additive explanations (SHAP) (29). This approach quantifies the contribution of each feature gene to the model output, thereby providing a reliable basis for understanding the key mechanisms underlying model predictions.

### Single-cell RNA sequencing analysis

To characterize the cellular expression patterns of the hub genes and further assess their association with EOPE, we analyzed a publicly available placental single-cell RNA-sequencing dataset comprising EOPE samples and gestational age-matched PTB controls using the 10× Genomics Chromium platform (30). The single-cell expression matrices and original cell-type annotations were obtained directly from the original study, in which quality control, clustering, and cell-type annotation had already been performed. Related annotated subclusters were merged into major cell populations for downstream analyses. The data were subsequently re-analyzed using Seurat (v5.3.0), with Harmony (v1.2.3) applied for batch correction and dimensionality reduction. Differential expression analysis, pseudotime trajectory analysis, and cell–cell communication analysis were performed using Seurat, Monocle2 (v2.36.0), and CellChat (v1.6.1), respectively.

### Quantitative real-time PCR (qRT-PCR)

This study was approved by the Institutional Ethics Committee of Shanghai First Maternity and Infant Hospital (No. 2019-034). Placental tissue samples were collected from women who delivered at Shanghai First Maternity and Infant Hospital. Written informed consent was obtained from all participants prior to enrollment. PE was diagnosed as new-onset hypertension after 20 weeks of gestation, defined as systolic blood pressure ≥140 mmHg and/or diastolic blood pressure ≥90 mmHg, accompanied by proteinuria and/or evidence of maternal organ dysfunction. EOPE cases were defined as PE diagnosed before 34 weeks of gestation. Gestational age-matched PTB placentas were used as controls. PTB controls were defined as spontaneous preterm deliveries without hypertensive disorders of pregnancy or other major maternal or fetal complications. Relevant clinical information was collected from medical records, and the baseline characteristics of the study participants are summarized in **Supplementary Table S3**. Total RNA was extracted using the Total RNA Extraction Kit (AG21023, Accurate Biology). The isolated RNA was then reverse-transcribed into complementary DNA (cDNA) using the TaKaRa Taq™ kit (R001A, Takara) in accordance with the manufacturer’s instructions. qRT-PCR was subsequently performed using SYBR, UNG Plus (AG11763, Accurate Biology) on the QuantStudio™3 Real-Time PCR System. The primer sequences used in this study are listed in **Supplementary Table S4**.

### Western blot

After collection of the cells and placental tissues, total protein was extracted using RIPA lysis buffer (P0013C, Beyotime). The lysates were centrifuged at 14,000 × g for 15 min at 4 °C, and the supernatants were collected for subsequent protein analysis. Protein concentrations were determined using a BCA Protein Assay Kit (20201ES86, Yeasen Biotechnology). Equal amounts of protein were separated by 12.5% SDS–PAGE and then transferred onto PVDF membranes. The membranes were blocked at room temperature in TBST buffer (Tris-buffered saline containing 0.1% Tween-20) supplemented with 5% non-fat milk. The membranes were then incubated overnight at 4 °C with primary antibodies at the dilutions recommended by the manufacturers, including anti-P21 (82669, Proteintech), anti-leptin (A1300, ABclonal), anti-GAPDH (P60037, ABclonal), and anti-β-actin (P60709, Cell Signaling Technology). GAPDH and β-actin were used as the internal loading control. After thorough washing, the membranes were incubated with an HRP-conjugated secondary antibody, specifically HRP-conjugated donkey anti-rabbit IgG (H+L) (1:10,000; 711-066-152, Jackson ImmunoResearch). Protein bands were finally visualized using ECL chemiluminescent substrate (P0018AM, Beyotime Biotechnology) according to the manufacturer’s instructions.

### Assay of senescence (S-A-β-gal staining)

Senescence-associated β-galactosidase (S-A-β-gal) activity in both cultured cells and frozen tissue samples was determined using the commercial cellular senescence S-A-β-gal staining kit (Cat. No. C0602, Beyotime Biotechnology). Briefly, treated cultured cells and frozen tissue sections were subjected to fixation and staining strictly in accordance with the manufacturer’s instructions.

### Immunohistochemistry analysis

Paraffin-embedded placental specimens were sectioned into 4 μm-thick slices using a Leica microtome. The obtained placental tissue sections were incubated overnight at 4 °C in a humidified incubation chamber with the primary antibody: anti-rabbit leptin (A1300, ABclonal). To guarantee the objectivity of the experimental results and reduce potential bias to the greatest extent, a blinded evaluation approach was adopted. For each placental sample, three representative visual fields were randomly chosen for microscopic observation and subsequent quantitative analysis.

### HCG measurement

After incubation, cell culture supernatants were harvested, and human chorionic gonadotropin (hCG) concentrations were quantified using the Human hCG ELISA Kit (Cat. No. R-M-H24543, Boerfu) in accordance with the manufacturer’s instructions.

### Statistical analysis

Comparisons between two groups were conducted using Student’s t test. Correlations between continuous variables were assessed using Spearman’s correlation analysis and P < 0.05 was considered statistically significant. For multiple testing correction, P values were adjusted using the Benjamini-Hochberg false discovery rate (FDR) method. Data are presented as the mean ± standard deviation (SD) from at least three independent experiments. GraphPad Prism (version 9.0.0) was used for data visualization and supplementary statistical analyses.

## Results

### 3.1 Global transcriptomic alterations in EOPE placentas

**Figure 1:**
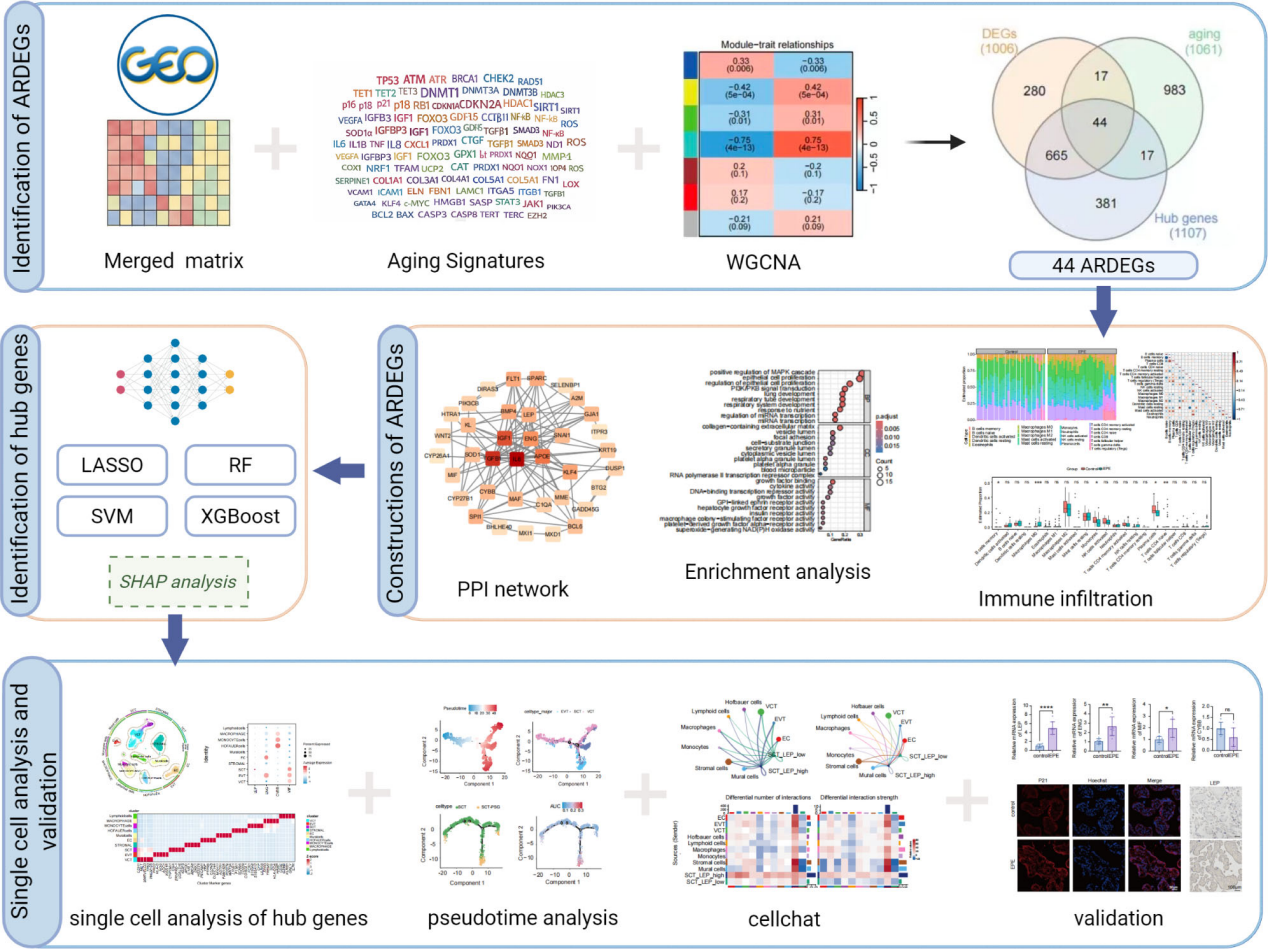
Flow chart of the study design. An overview of the analytical workflow including data collection, WGCNA, machine learning, single-cell, and trajectory analyses and validation.

**Figure 2:**
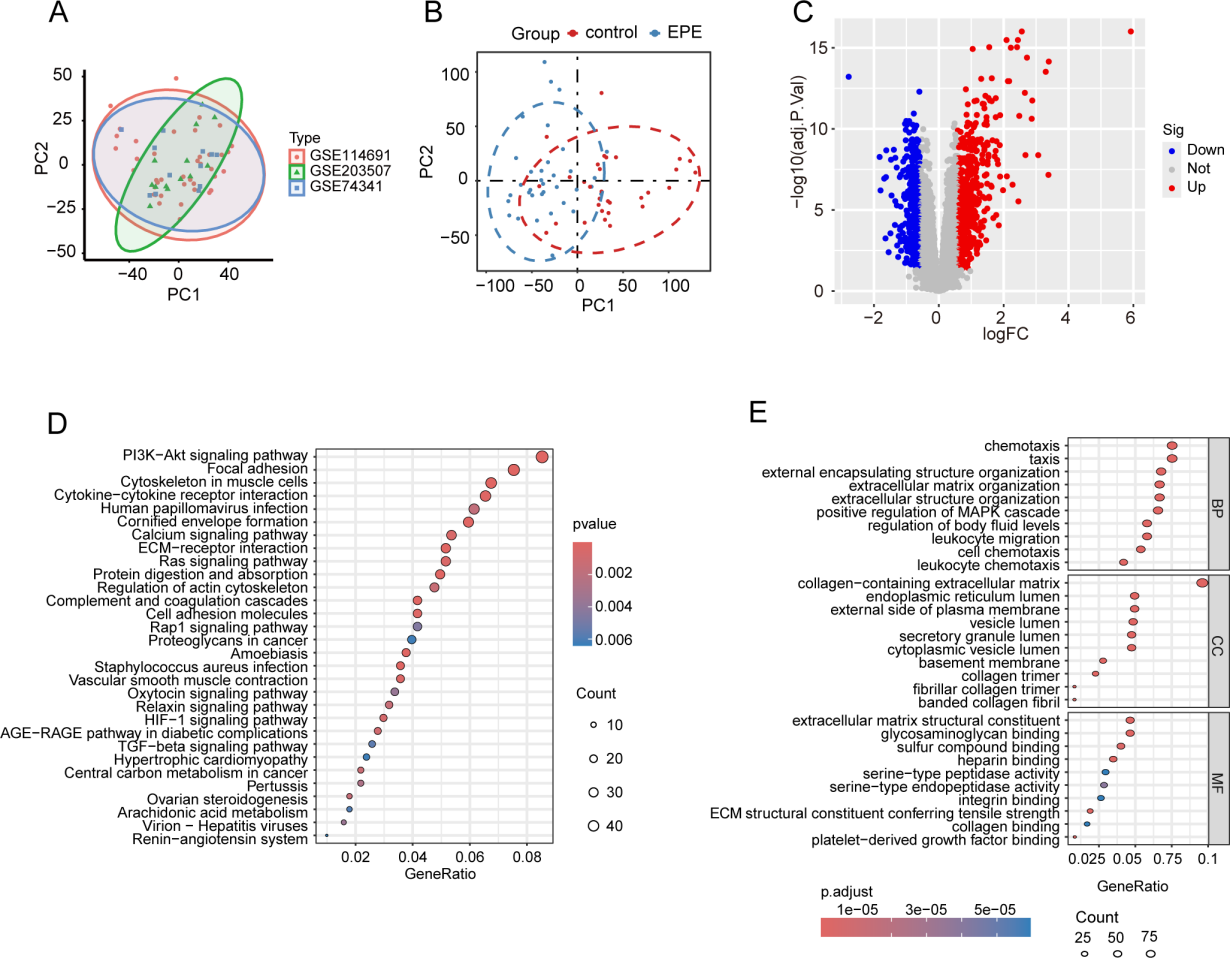
Global transcriptomic alterations in EOPE placentas. **(A)** PCA of the integrated placental transcriptomic datasets before batch correction. **(B)** PCA of EOPE and gestational age-matched PTB placentas after normalization and batch correction. **(C)** Volcano plot showing DEGs between EOPE and PTB placentas. **(D)** Kyoto Encyclopedia of Genes and Genomes (KEGG) enrichment analysis of the DEGs. **(E)** Gene Ontology (GO) enrichment analysis of the DEGs across biological process (BP), cellular component (CC) and molecular function (MF).

We have drawn a specific technology roadmap for this article (**Figure 1**). To characterize transcriptomic alterations in EOPE, we integrated three placental transcriptomic datasets (GSE114691, GSE203507, and GSE74341). Before batch correction, samples showed clear dataset-dependent separation (**Figure 2A**). After normalization and batch correction, EOPE placentas remained clearly separated from gestational age-matched PTB controls, indicating robust disease-associated transcriptional changes (**Figure 2B**). Differential expression analysis revealed widespread gene dysregulation in EOPE placentas (**Figure 2C**). KEGG analysis revealed significant enrichment in the PI3K–Akt signaling pathway, focal adhesion, cytokine-cytokine receptor interaction, ECM-receptor interaction and the AGE-RAGE signaling pathway (**Figure 2D**). GO analysis further highlighted enrichment in leukocyte chemotaxis, extracellular matrix organization, collagen-containing extracellular matrix, and growth factor binding (**Figure 2E**). Together, these findings indicate that EOPE is characterized by inflammatory activation, extracellular matrix remodeling, and dysregulated stress-response signaling, with several enriched pathways also implicating aging-related molecular processes.

### 3.2 Identification of senescence-related hub candidate genes in EOPE

**Figure 3:**
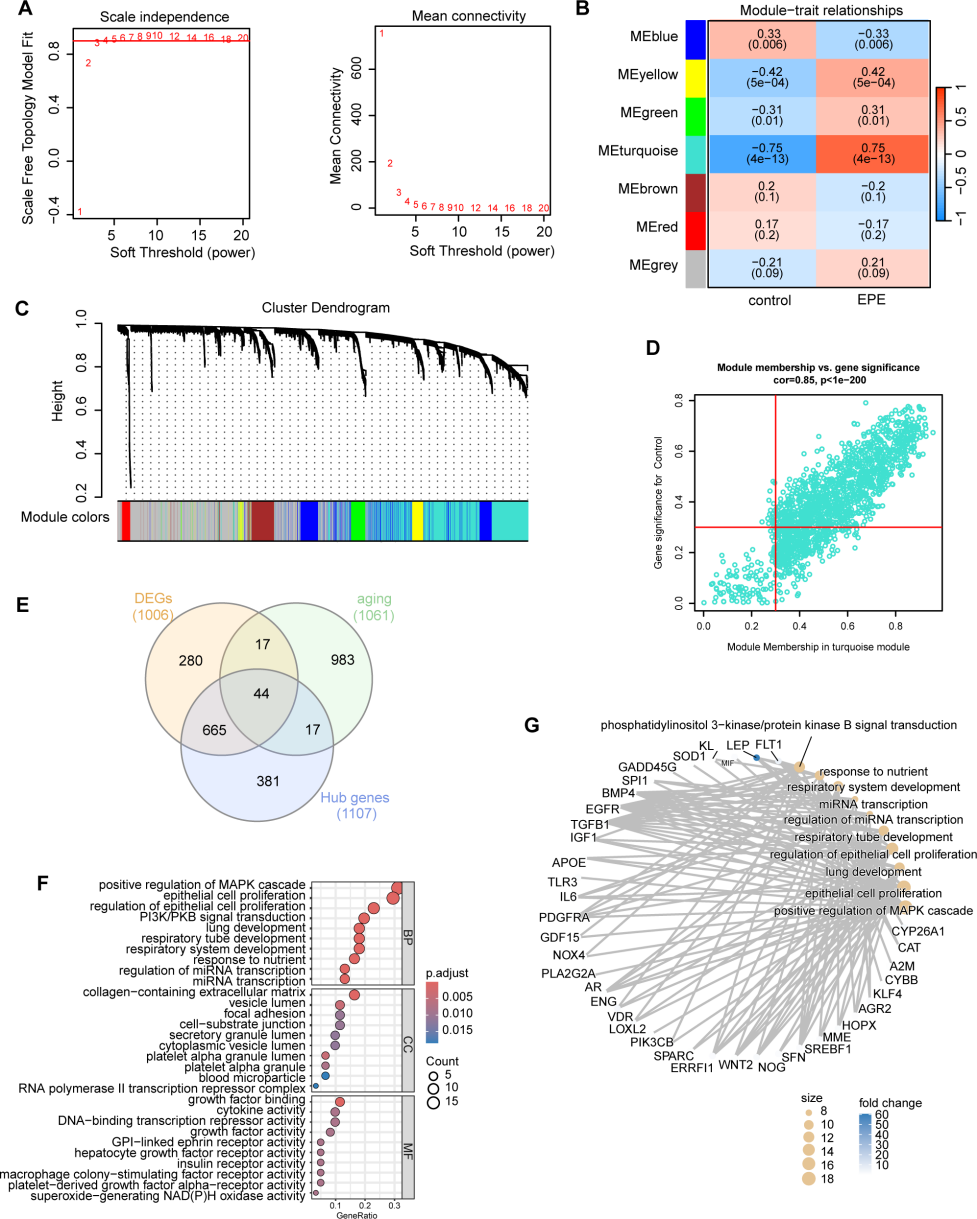
Identification of senescence-related genes associated with EOPE. **(A)** Scale-free topology fit index and mean connectivity used to determine the soft-thresholding power for WGCNA. **(B)** Module–trait relationships between co-expression modules and EOPE status. **(C)** Hierarchical clustering dendrogram showing module assignment. **(D)** Correlation between module membership and gene significance in the turquoise module. **(E)** Venn diagram showing the overlap among DEGs, SRGs and EOPE-associated module genes, identifying 44 SRDEGs. **(F)** GO enrichment analysis of the 44 SRDEGs. **(G)** Gene–pathway interaction network highlighting representative SRDEGs and enriched biological processes.

To identify gene modules associated with EOPE, WGCNA was performed using a soft-thresholding power of 8 to construct a scale-free co-expression network, from which seven modules were obtained after merging closely related modules (**Figures 3A–C**). Among them, the turquoise module showed the strongest correlation with EOPE (cor = 0.85; **Figure 3D**; **Supplementary Figure S2A**). Intersecting genes from this module with DEGs and SRGs identified 44 SRDEGs (**Figure 3E**; **Supplementary Table S2**). Functional enrichment analysis showed that these genes were mainly involved in epithelial cell proliferation, PI3K/PKB signaling, MAPK cascade and nutrient response. Gene–pathway network analysis further highlighted representative genes, including LEP, ENG, MIF, PIK3CB, EGFR, TGFB1, IL6, CYBB, and GADD45G, which were closely connected to these enriched processes (**Figures 3F, G**). Together, these results suggest that senescence-related molecular alterations in EOPE are associated with dysregulated proliferative and stress-response signaling. Then, A PPI network of the 44 ARDEGs was constructed using the STRING database (**Supplementary Figure S2D**). The MCODE plugin was employed to identify gene cluster modules, while the CytoHubba plugin was utilized to assign scores to the selected genes (**Supplementary Figure S2E**).

### 3.3 Consensus clustering reveals two senescence-related EOPE subtypes

**Figure 4:**
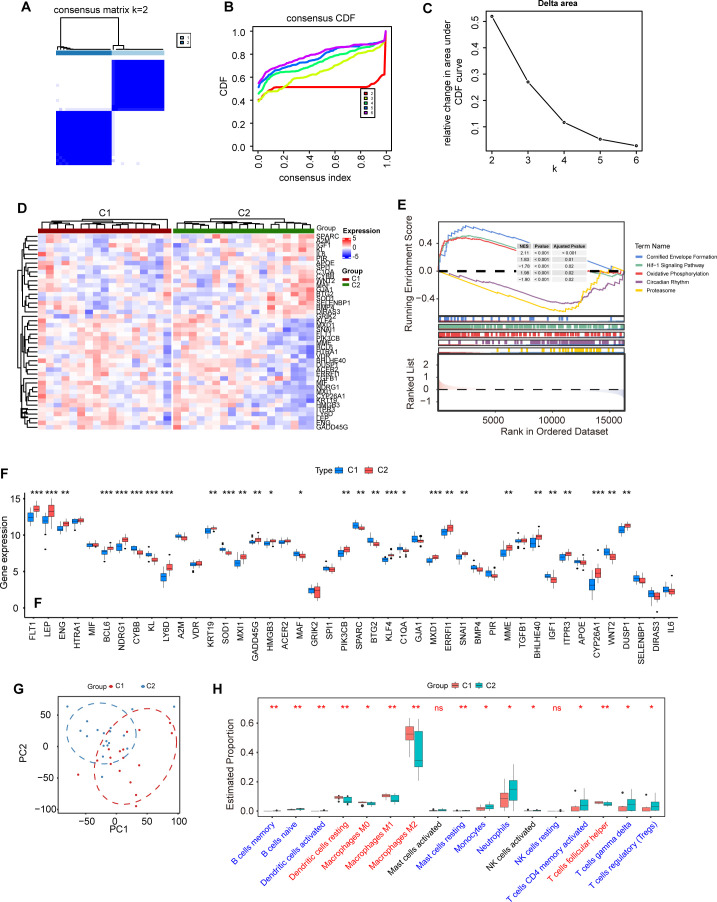
Consensus clustering identifies two senescence-related EOPE subtypes. **(A)** Consensus matrix for clustering at *k *= 2. **(B)** Cumulative distribution function (CDF) plot for consensus clustering. **(C)** Relative change in area under the CDF curve for different cluster numbers. **(D)** Heatmap showing the expression pattern of the 44 SRDEGs across the two molecular subtypes, C1 and C2. **(E)** Gene set enrichment analysis comparing the two subtypes. **(F)** Expression differences of the 44 SRDEGs between control and EOPE groups. **(G)** PCA plot based on the 44 SRDEGs. **(H)** Comparison of immune-cell infiltration between C1 and C2.

To further investigate heterogeneity within the senescence-associated transcriptional program, consensus clustering was performed using the 44 SRDEGs. The optimal clustering solution was obtained at k = 2, defining two molecular subtypes, C1 and C2 (**Figures 4A–C**). The two clusters showed distinct SRDEG expression patterns (**Figure 4D**), and gene set enrichment analysis indicated differential pathway activity between them, including cornified envelope formation, HIF-1 signaling, oxidative phosphorylation, circadian rhythm, and proteasome-related pathways (**Figure 4E**). Expression profiling of the 44 SRDEGs further showed marked differences between control and EOPE samples (**Figure 4F**), and PCA based on these genes separated the two clinical groups (**Figure 4G**). Immune infiltration analysis also demonstrated significant differences between C1 and C2 across multiple immune-cell populations (**Figure 4H**), indicating that senescence-related EOPE subtypes differ in both molecular features and immune contexture.

### 3.4 Identification of robust candidate hub genes using machine learning

**Figure 5:**
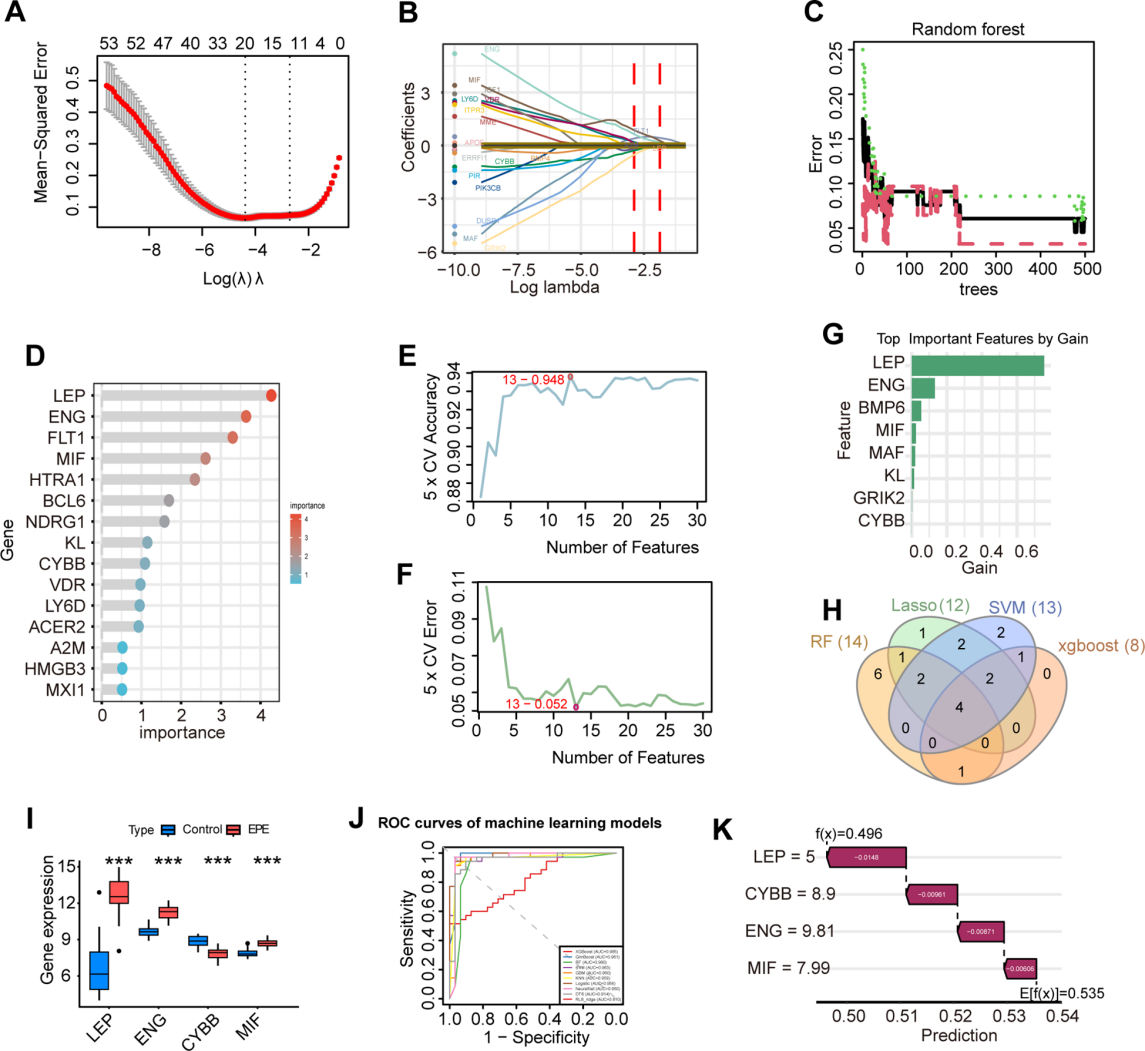
Machine learning prioritizes stable candidate hub genes in EOPE. **(A, B)** LASSO regression for feature selection, including cross-validation error and coefficient profiles. **(C, D)**. Random forest analysis showing model error across trees and variable importance ranking. **(E, F)** SVM-RFE showing classification accuracy and cross-validation error at different feature numbers. **(G)** Feature importance ranking derived from XGBoost analysis. **(H)** Venn diagram showing the overlap among genes selected by LASSO, random forest, SVM-RFE and XGBoost, identifying four stable hub genes. **(I)** Expression of LEP, ENG, CYBB and MIF in EOPE and PTB placentas. Bars represent mean ± SD. **(J)** Receiver operating characteristic (ROC) curves of 10 machine-learning models for EOPE classification. **(K)** Representative SHAP force plot showing the contribution of each hub gene to prediction in an individual sample.

To further identify robust candidate genes from the 44 SRDEGs, four machine learning algorithms were applied, including LASSO regression, RF, SVM-RFE, and XGBoost. LASSO regression identified 12 candidate genes at the optimal lambda value (**Figures 5A, B**), RF prioritized genes according to their variable importance scores (**Figures 5C, D**), SVM-RFE identified an optimal feature set of 13 genes (**Figures 5E, F**), and XGBoost selected 8 key variables according to gain values (**Figure 5G**). By intersecting the results of four machine learning algorithms, we identified LEP, ENG, MIF and CYBB as senescence-related hub genes associated with EOPE (**Figure 5H**). Further analysis revealed that LEP, ENG and MIF were significantly upregulated in EOPE placentas, whereas CYBB was significantly downregulated (**Figure 5I**).

### 3.5 Machine-learning prioritization of candidate senescence-associated genes and SHAP-based feature interpretation

To prioritize key senescence-associated genes linked to EOPE, we evaluated ten mainstream machine learning models using the merged dataset. Among them, XGBoost achieved the best performance (AUC of 0.985; **Figure 5J**) and was therefore selected for subsequent interpretation. SHAP analysis using the permshap function showed that LEP contributed most to the model, followed by ENG, MIF, and CYBB (**Supplementary Figure S4A–C**). At the individual-sample level, a representative SHAP force plot further demonstrated that LEP had the greatest impact on prediction outcome (**Figure 5K**; **Supplementary Figure S4D**), suggesting a central role in model performance. In the external validation cohort GSE44711, LEP, ENG, and MIF remained significantly upregulated, whereas CYBB showed a consistent downregulation trend (**Supplementary Figure S4E**), supporting the robustness of these hub genes. Although derived from placental tissue and not directly applicable to noninvasive testing, these biomarkers provide mechanistic insight into EOPE and may serve as a stable panel for early disease recognition.

### 3.6 Immune dysregulation of the placental microenvironment in EOPE

**Figure 6:**
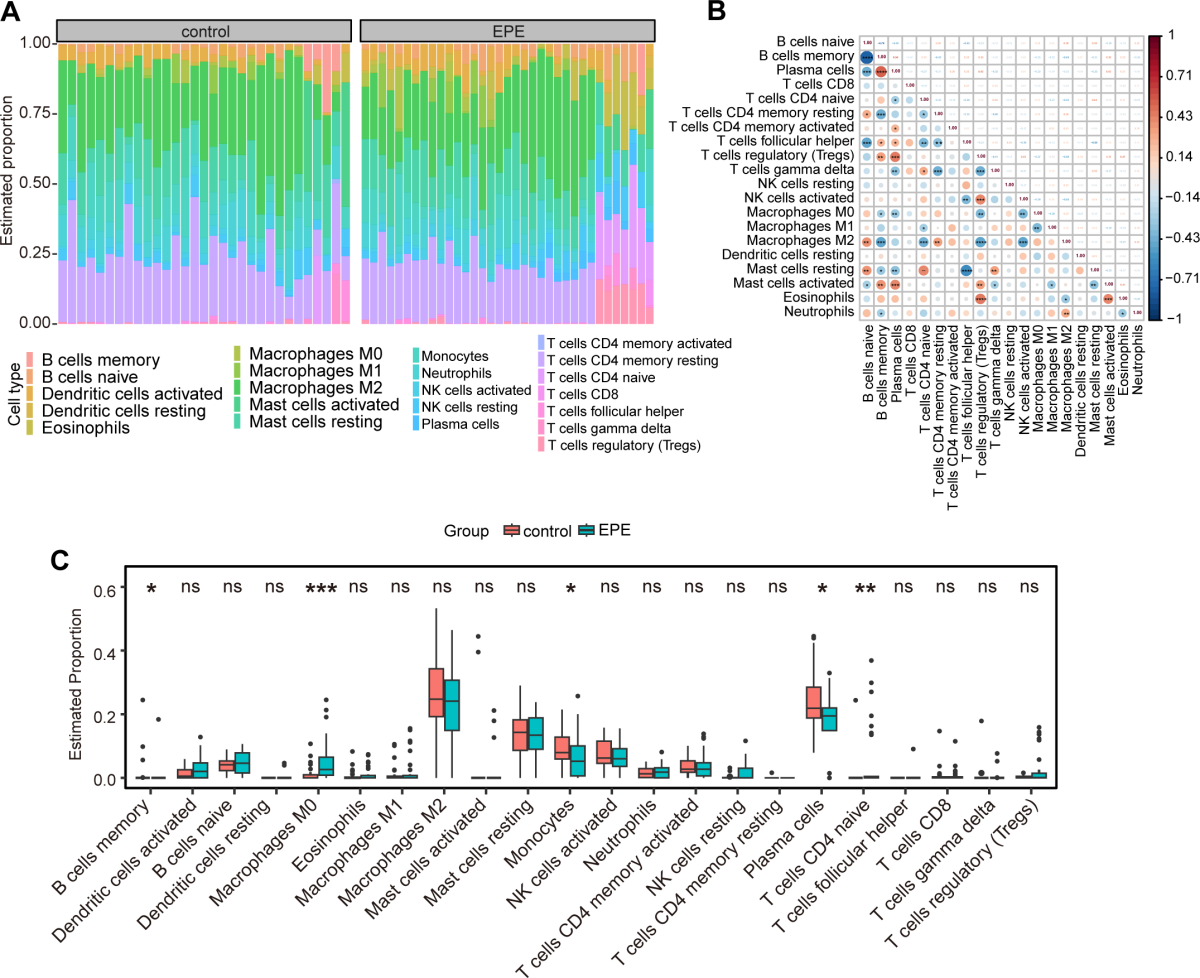
EOPE placentas exhibit immune microenvironment remodeling. **(A)** Stacked bar plot showing the estimated proportions of immune-cell subsets in PTB and EOPE placentas based on CIBERSORT analysis. **(B)** Correlation matrix of infiltrating immune-cell populations. **(C)** Comparison of immune-cell infiltration between PTB and EOPE placentas across immune-cell subsets.

Emerging evidence suggests that senescence-associated secretory phenotype (SASP) factors can modulate immune-cell recruitment and activation, thereby contributing to tissue inflammation and microenvironmental remodeling (31, 32). To investigate the potential association between senescence and immune dysregulation in EOPE, CIBERSORT revealed M2 macrophages, resting CD4 memory T cells, monocytes, and neutrophils as the predominant immune cell populations in the placenta (**Figure 6A**). EOPE placentas showed selective alterations in memory B cells, M0 macrophages, monocytes, and naive CD4 T cells, with M0 macrophages exhibiting the greatest increase (**Figure 6C**). Correlation analysis revealed coordinated changes among immune subsets, including a negative correlation between naive B cells and memory B cells and a positive correlation between memory B cells and plasma cells, suggesting broader remodeling of the placental immune network in EOPE (**Figure 6B**). These results indicate that EOPE is characterized by selective immune dysregulation that may contribute to local inflammation and placental microenvironmental imbalance.

### 3.7 Single-cell analysis of SRGs

**Figure 7:**
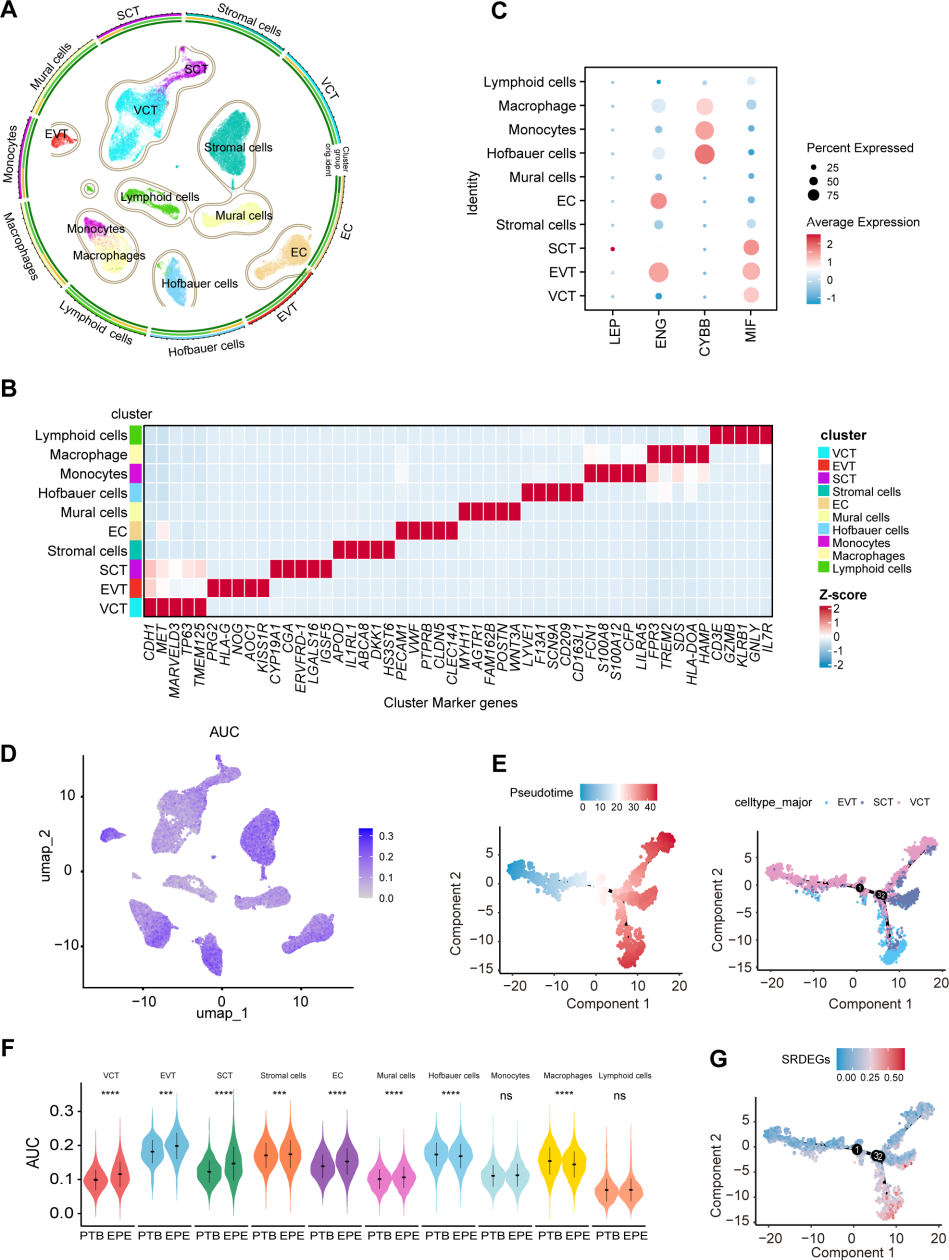
Single-cell analysis localizes hub genes and senescence signals to trophoblast populations. **(A)** UMAP showing the major placental cell populations. **(B)** Heatmap of canonical marker genes used for cell-type annotation. **(C)** Dot plot showing the expression distribution of LEP, ENG, CYBB and MIF across major placental cell populations. Dot size indicates the fraction of expressing cells and colour indicates average expression level. **(D)** UMAP showing AUCell-based senescence scores across placental cell populations. **(E)** Trajectory analysis of trophoblast populations coloured by pseudotime and cell state. **(F)** Comparison of senescence AUC scores between PTB and EOPE across major cell populations. **(G)** Projection of SRDEG activity onto the trophoblast trajectory.

We analyzed a published single-cell RNA-sequencing dataset to define the cell type-specific expression of the hub genes and to identify the principal placental cell populations associated with senescence-related phenotypes. UMAP analysis identified the major placental cell populations, including villous cytotrophoblasts (VCT), extravillous trophoblasts (EVT), SCT, stromal cells, endothelial cells (EC), mural cells, Hofbauer cells, monocytes, macrophages, and lymphoid cells (**Figure 7A**). Cell identities were assigned based on canonical marker genes (**Figure 7B**). Single-cell expression mapping showed that LEP was predominantly enriched in SCT, ENG in EVT and EC, CYBB in monocytes, macrophages, and Hofbauer cells, and MIF across trophoblast and immune cell populations (**Figure 7C**). AUCell scoring based on the SRDEG set showed that senescence signals were broadly distributed across placental cell types, with the strongest enrichment in the trophoblast lineage (**Figure 7D**). Compared with PTB controls, EOPE placentas showed significantly higher senescence scores in VCT, EVT, SCT, stromal cells, EC, Hofbauer cells, monocytes, and macrophages (**Figure 7F**).

**Figure 8:**
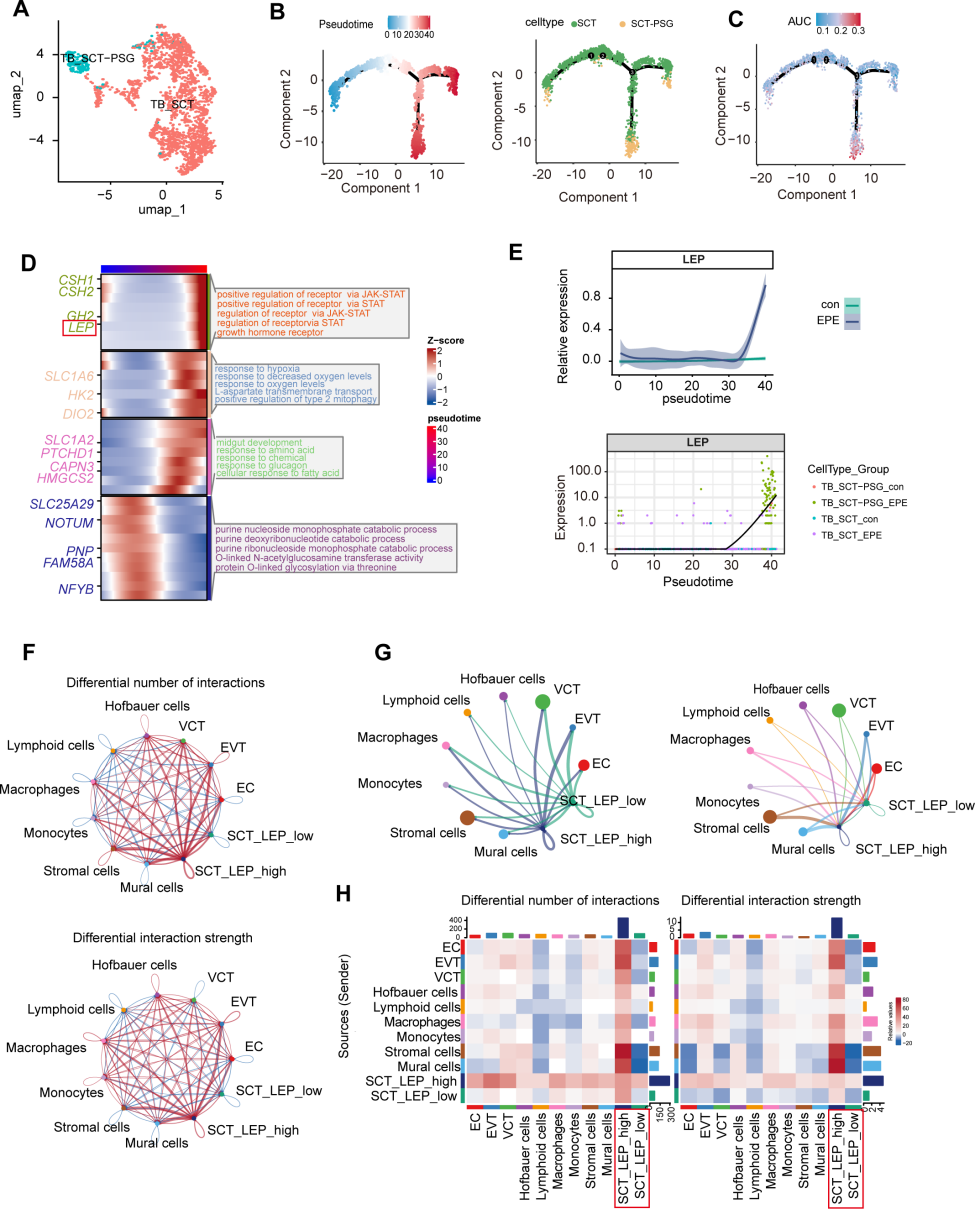
LEP marks a senescence-associated and communication-active SCT state. **(A)** UMAP plot showing SCT-related subclusters, including TB_SCT and TB_SCT-PSG. **(B)** Pseudotime trajectory of SCT-related cells. **(C)** Projection of senescence AUC scores onto the SCT trajectory. **(D)** Heatmap of pseudotime-dependent gene modules and representative enriched biological processes across SCT differentiation. **(E)** Dynamic expression of LEP along pseudotime shown as smoothed curves and single-cell scatter plots. **(F)** Differential interaction network between SCT_LEP_high and SCT_LEP_low states based on the number of cell–cell interactions. **(G)** Differential interaction network between SCT_LEP_high and SCT_LEP_low states based on interaction strength. **(H)** Heatmaps summarizing differential interaction number and interaction strength among placental cell populations.

Pseudotime analysis showed that senescence-associated transcriptional activity increased progressively along trophoblast differentiation (**Figures 7E, G**). Admati et al. classified SCTs into two subtypes, SCT and SCT-PSG. Among these, SCT-PSG represented a more mature secretory and endocrine state, characterized by high expression of PSG family genes and KISS1 (30). Notably, EOPE-associated abnormalities were predominantly concentrated within this syncytial lineage (**Figures 8A, B**). Consistently, our analysis showed that senescence activity increased along the SCT trajectory and peaked in late TB_SCT-PSG, indicating that EOPE-associated senescence is most prominent in terminally mature SCT (**Figure 8C**). Pseudotime-driving genes in late SCT were mainly enriched in JAK-STAT signaling, hypoxia response, metabolic pathways, and protein glycosylation, indicating marked stress, inflammatory, and endocrine remodeling in this state (**Figure 8D**).

### 3.8 Senescence-associated LEP-high SCT reshape the placental signaling microenvironment in EOPE

Among the identified hub genes, LEP showed the strongest association with SCTs, with expression increasing progressively along trophoblast pseudotime and reaching its highest levels in EOPE, particularly within the TB_SCT-PSG subpopulation (**Figure 8E**). Other hub genes, including ENG, CYBB, and MIF, also displayed pseudotime-dependent expression dynamics (**Supplementary Figures S7B, C**), indicating that hub-gene dysregulation is tightly linked to trophoblast cell state. To define the signaling features of LEP-high SCTs, we stratified SCTs into SCT_LEP_high and SCT_LEP_low subsets and performed CellChat analysis. LEP-high SCTs exhibited marked rewiring of intercellular communication, with substantial changes in both interaction number and interaction strength across multiple placental cell populations (**Figures 8F, G**). Consistently, EOPE placentas showed global remodeling of communication networks relative to PTB controls, including altered interaction strength, interaction number, and dominant sender–receiver patterns (**Figure 8H**; **Supplementary Figures S6A–D**). Genes upregulated in LEP-high SCTs were enriched in endocrine- and hormone-response pathways, including growth hormone receptor signaling and peptide hormone response (**Supplementary Figure S6E**), supporting a specialized trophoblast state with enhanced stress responsiveness and signaling activity. In parallel, MIF signaling was also increased among SCTs, monocytes, macrophages, and stromal cells in EOPE, with strengthened interactions involving MIF-(CD74+CXCR4) and MIF-(CD74+CD44) (**Supplementary Figures S6F–H**). Together, these findings suggest that senescence-associated trophoblast states contribute to EOPE placental microenvironment remodeling through coordinated endocrine and inflammatory signaling programs.

### 3.9 Experimental validation supports a senescence-associated role of LEP in EOPE

**Figure 9:**
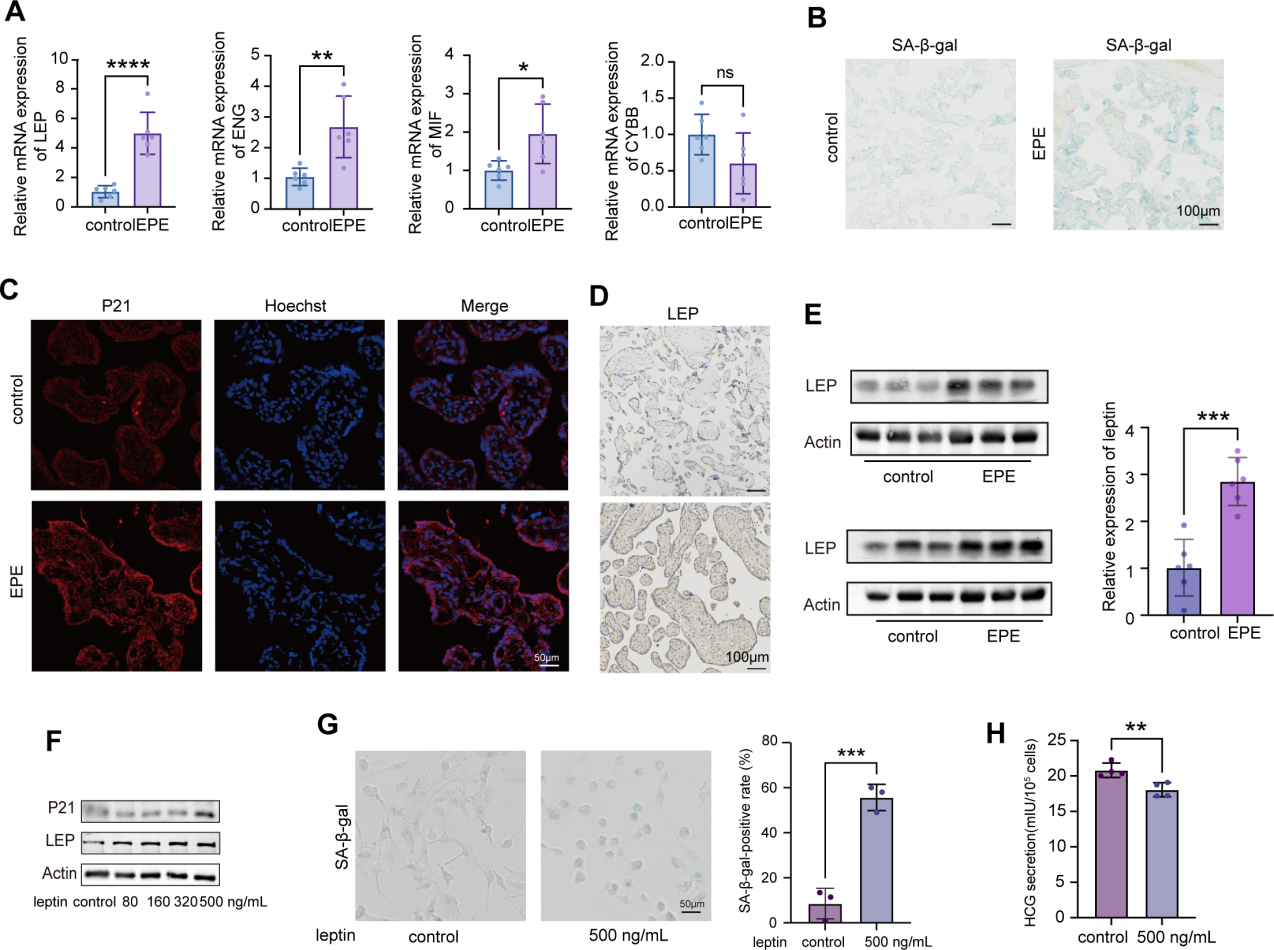
Experimental validation supports a senescence-associated role of LEP in EOPE. **(A)** qRT–PCR analysis of LEP, ENG, MIF and CYBB mRNA expression in PTB and EOPE placentas. n = 6 biologically independent placentas per group. Bars represent mean ± SD. **(B)** Representative images of senescence-associated β-galactosidase (SA-β-gal) staining. **(C)** Representative immunofluorescence images of P21 in PTB and EOPE placentas. Nuclei were counterstained with Hoechst. **(D)** Representative images of LEP immunohistochemistry in PTB and EOPE placentas. Scale bars, 100 μm. n = 6 biologically independent placentas per group. **(E)** Representative western blot showing LEP protein expression in PTB and EOPE placentas. n = 6. **(F)** Western blot analysis of P21 and LEP protein expression in BeWo cells treated with increasing concentrations of leptin. **(G)** Representative SA-β-gal staining images (left) and quantification of SA-β-gal-positive cells (right) in BeWo cells treated with leptin. n = 3 biologically independent experiments. Bars represent mean ± SD. **(H)** Detection of hCG in cell culture supernatant by ELISA after 24 h of leptin treatment.

To validate the transcriptomic findings, we examined hub gene expression in placental tissues from EOPE and PTB controls. qRT-PCR showed that LEP, ENG, and MIF mRNA levels were significantly increased in EOPE placentas, whereas CYBB showed no significant difference (**Figure 9A**). Placental senescence was also increased in EOPE tissues, as indicated by increased SA-β-gal staining (**Figure 9B**) and enhanced P21 immunofluorescence (**Figure 9C**) compared with PTB controls. LEP immunohistochemistry further showed stronger staining in EOPE placentas (**Figure 9D**), and western blot analysis confirmed increased LEP protein expression in EOPE tissues (**Figure 9E**). To test whether leptin directly promotes trophoblast senescence, BeWo cells were treated with increasing concentrations of leptin. Leptin treatment induced LEP and P21 protein expression in a dose-dependent manner (**Figure 9F**). High-dose leptin significantly increased the proportion of SA-β-gal-positive cells (**Figure 9G**), supporting a pro-senescent effect of leptin in trophoblasts. Consistently, leptin treatment induced senescence-associated changes, as evidenced by P21 upregulation, increased SA-β-gal positivity, and a slight reduction in hCG secretion from BeWo with leptin treatment (**Figure 9I**).

## Discussion

EOPE is a severe placenta-mediated disorder, yet the molecular basis linking placental dysfunction to premature senescence remains incompletely understood (31, 33). Although cellular senescence is increasingly recognized as a contributor to PE, physiological placental aging during late gestation poses a major challenge for defining disease-specific senescence programs (34, 35). By using PTB placentas as controls, we minimized this confounding effect and systematically characterized the senescence-associated molecular and cellular landscape of EOPE at both bulk and single-cell resolution. Functional enrichment analysis revealed that the 44 SRDEGs were predominantly enriched in pathways regulating epithelial cell proliferation, PI3K–Akt signaling, MAPK cascade, focal adhesion, extracellular matrix organization, and hypoxia response. These pathways are critically implicated in trophoblast dysfunction, impaired spiral artery remodeling, and placental hypoperfusion, which represent core initiating events in EOPE pathogenesis (36, 37). The convergence of SRDEGs and stress- inflammatory signaling cascades further supports that EOPE placentas exhibit a state of pathologically accelerated senescence rather than normal gestational aging (38).

In this study, we integrated bulk transcriptomics, WGCNA, machine learning and other approaches to identify 44 SRDEGs associated with EOPE. Four machine learning strategies were further applied to recognize LEP, ENG, MIF, and CYBB as core molecules linked to EOPE-related placental senescence. ENG is a key regulator of placental vascular remodeling, and increased placental and circulating endoglin, particularly soluble endoglin, has been widely implicated in EOPE and maternal endothelial dysfunction (39, 40). We further verified ENG as a core molecule in EOPE-related placental senescence, suggesting that it may exacerbate placental dysfunction not only by regulating vascular remodeling but also by mediating placental senescence, thus refining its underlying mechanism. Oxidative stress is a well−established driver of placental senescence in PE (41). CYBB encodes NOX2, the catalytic subunit of the NADPH oxidase complex, and its dysregulated expression disrupts redox homeostasis (42–44). Although CYBB was identified as a hub senescence-related gene in the integrated transcriptomic analyses, its differential expression was not confirmed in our clinical placental samples. This discrepancy may reflect the limited sample size, biological heterogeneity among EOPE patients, and the cell-type-specific expression pattern of CYBB, which may be obscured in bulk tissue analyses. Therefore, the association of CYBB with EOPE and placental senescence requires further validation in larger sample sets and at single-cell resolution. MIF encodes macrophage migration inhibitory factor, a pleiotropic pro-inflammatory cytokine that has been confirmed to be involved in innate immune regulation, placental development, and maternal-fetal immune adaptation (45, 46). Our study demonstrated that MIF is not only expressed in immune cells but also widely distributed in trophoblast cells and stromal cells. In the placenta of EOPE, MIF signaling transduction is significantly enhanced among SCTs, monocytes, macrophages, and stromal cells. Specifically, the interactions between MIF and its functional receptor CD74, as well as the chemokine receptor CXCR4, are significantly strengthened. Meanwhile, the MIF-(CD74+CXCR4) related signaling pathway is obviously activated, which further amplifies the placental inflammatory response and is closely associated with the pathophysiological process of EOPE. The consistent dysregulation of these genes across datasets further validates the credibility of our senescence-related gene screening framework.

Single-cell RNA sequencing showed that EOPE was associated with broad activation of senescence-related programs across multiple placental cell populations, including trophoblasts, stromal cells, endothelial cells, and immune cells. Recent studies suggest that senescence-like programs are an integral component of normal placental maturation and trophoblast differentiation (47). However, the increased senescence signatures observed in EOPE compared should not be interpreted simply as a generalized increase in placental aging. Instead, accumulating evidence suggests that disruption of normally regulated senescence programs may contribute to the development of PE (48). Consistent with these findings, our single-cell and pseudotime analyses revealed that EOPE may be associated with premature or dysregulated activation of senescence programs that normally accompany trophoblast differentiation and SCT maturation.

Among the four hub genes, LEP emerged as the most prominent candidate associated with placental senescence in EOPE. Accumulating evidence has consistently shown that leptin levels are markedly increased in PE placenta and closely linked to placental hypoxia, oxidative stress, inflammatory activation, and endothelial dysfunction (49, 50). Further studies in human trophoblasts have suggested that leptin is not merely a passive biomarker of placental stress, but actively regulates cytotrophoblast invasion in a gestational age and dose−deopendent manner (51). Meanwhile, leptin amplifies placental inflammation by inducing IL-6 in Hofbauer cells via the ERK1/2 MAPK pathway, with increased LEPR and IL-6 immunoreactivity in preeclamptic placentas (52). Recently, integrated transcriptomic and machine-learning analyses have identified multiple EOPE-associated genes, including LEP, which may be involved in immune dysregulation and alterations of the placental immune microenvironment (53, 54). However, whether LEP participates in the pathogenesis of EOPE through the pathological mechanism of inducing placental senescence remains unclear. In the present study, we identified a critical role of LEP in driving excessive placental senescence in EOPE. LEP was enriched in the mature secretory SCT-PSG subset and gradually upregulated along the pseudotime trajectory of trophoblast differentiation, indicating a close association with aberrant syncytial maturation and stress accumulation. Concomitantly, profound rewiring of intercellular communication networks across placental cell populations suggests that LEP overexpression in SCT modulates intercellular crosstalk, disrupting the placental microenvironment and contributing to EOPE pathogenesis. Consistent with our *in vitro* findings, LEP may contribute to SCT senescence and cellular dysfunction in EOPE. Although increased hCG expression has been reported in PE placentas, elevated hCG levels do not necessarily indicate preserved trophoblast function (55). Instead, hCG dysregulation may reflect placental stress and pathological alterations, including mitochondrial dysfunction and inflammation (56). Therefore, the effects observed in our LEP-induced senescence model likely reflect the functional consequences of cellular senescence rather than the absolute hCG levels observed in PE placentas. Finally, the potential translational value of LEP is further supported by a recent cell−free RNA (cfRNA) study, which identified circulating LEP as one of the strongest predictors of PE complicated by fetal growth restriction (57).

Several limitations of this study should be acknowledged. First, although we integrated three EOPE datasets and included an independent validation dataset, the overall sample size remains relatively limited, and the findings require further validation in larger cohorts. Second, gestational age-matched PTB placentas were used as controls to minimize confounding effects related to gestational age, placental maturation, and physiological senescence. However, PTB placentas may exhibit biological alterations distinct from uncomplicated pregnancies, which could influence the observed transcriptional differences. Finally, the number of clinical samples used for experimental validation was relatively small, and additional studies are needed to confirm the stability and clinical relevance of the identified senescence-related hub genes.

In summary, by using gestational age-matched PTB controls to exclude the interference of physiological aging, our study systematically reveals that EOPE placentas present a global multi-lineage pathological senescence state, and identifies four core hub genes involved in regulating this process. We further clarify that LEP, which is specifically enriched in the mature secretory SCT-PSG subset, drives trophoblast senescence and reshapes the placental microenvironment. These findings provide a novel mechanistic framework for understanding EOPE pathogenesis from the perspective of placental senescence, and offer a theoretical basis for targeting placental senescence to intervene in EOPE progression.

## Data Availability

The original contributions presented in the study are included in the article/**Supplementary Material**. Further inquiries can be directed to the corresponding authors.

## Supplementary material

Supplementary Figure 1 Additional preprocessing and enrichment results for the integrated bulk transcriptomic datasets. **(a)** PCA of the three placental transcriptomic datasets before batch correction. **(b)** Distribution of normalized expression values across samples after preprocessing. **(c)** Circular GO enrichment plot of DEGs across BP, CC and MF categories.

Supplementary Figure 2 Additional WGCNA module-level enrichment analysis. **(a)** Correlation between module membership and gene significance in the turquoise module. **(b)** KEGG enrichment analysis of genes in the turquoise module. **(c)** Circular KEGG enrichment plot of turquoise-module genes. **(d)** PPI network showing the interaction relationships among the 44 ARDEGs. **(e)** A key module with 10 genes as the key gene was screened by MCODE

Supplementary Figure 3 Functional differences between the two senescence-related EOPE subtypes. **(a)** GO enrichment analysis of subtype C1. **(b)** GO enrichment analysis of subtype C2.

Supplementary Figure 4 SHAP interpretation and external validation of hub genes. **(a)** Mean absolute SHAP values of the four hub genes in the final predictive model. **(b)** SHAP summary plot showing the overall contribution of each hub gene across samples. **(c)** SHAP dependence plots showing the relationship between gene expression and SHAP values for LEP, ENG, MIF and CYBB. **(d)** Representative SHAP force plot illustrating feature contributions to model prediction. **(e)** Expression of LEP, ENG, CYBB and MIF in the external validation cohort. Bars represent mean ± SD.

Supplementary Figure 5 Hub-gene expression across major placental cell populations Violin plots showing the expression of LEP, ENG, CYBB and MIF across major placental cell populations in PTB and EOPE samples.

Supplementary Figure 6 Additional CellChat analysis and functional enrichment of LEP-high SCT cells. **(a)** Differential interaction network based on interaction number between EOPE and PTB placentas. **(b)** Differential interaction network based on interaction strength between EOPE and PTB placentas. **(c)** Dominant sender and receiver analysis comparing EOPE and PTB placentas. **(d)** Heatmaps showing overall signalling patterns in PTB and EOPE placentas. **(e)** GO biological process enrichment analysis of genes upregulated in LEP-high SCT cells. **(f)** MIF signalling pathway networks. **(g)** Dot plot showing the interaction probability of MIF ligand–receptor pairs. **(h)** Comparison of MIF signalling pathway networks between PTB and EOPE placentas.

Supplementary Figure 7 Additional pseudotime analysis of EOPE and PTB trophoblasts. **(a)** Distribution of PTB and EOPE trophoblast cells along the pseudotime trajectory. **(b)** Smoothed expression dynamics of ENG, CYBB and MIF along pseudotime. **(c)** Single-cell scatter plots showing the expression of CYBB, ENG and MIF along pseudotime.
