# Supplementary figures and images for: Integrative bioinformatics analysis identifies placental senescence-associated signatures in early-onset preeclampsia
Source: Front Endocrinol (Lausanne). 2026 Jul 8;17:1863608. doi: 10.3389/fendo.2026.1863608 (PMC13388174; doi:10.3389/fendo.2026.1863608)

Fig.S1

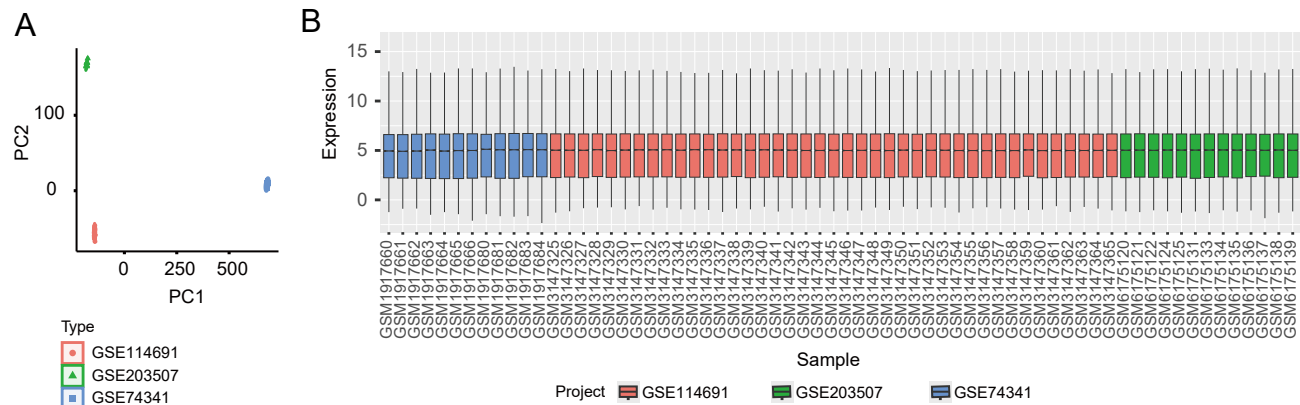

C

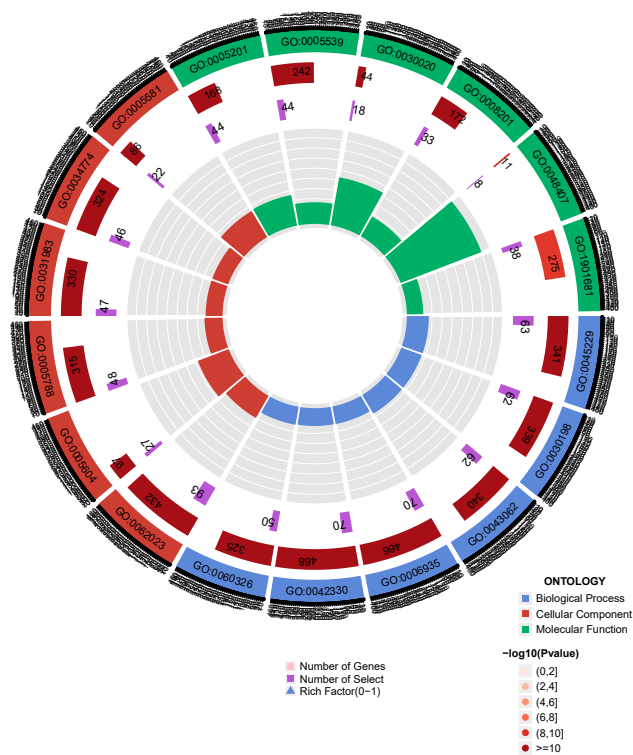

Fig.S2

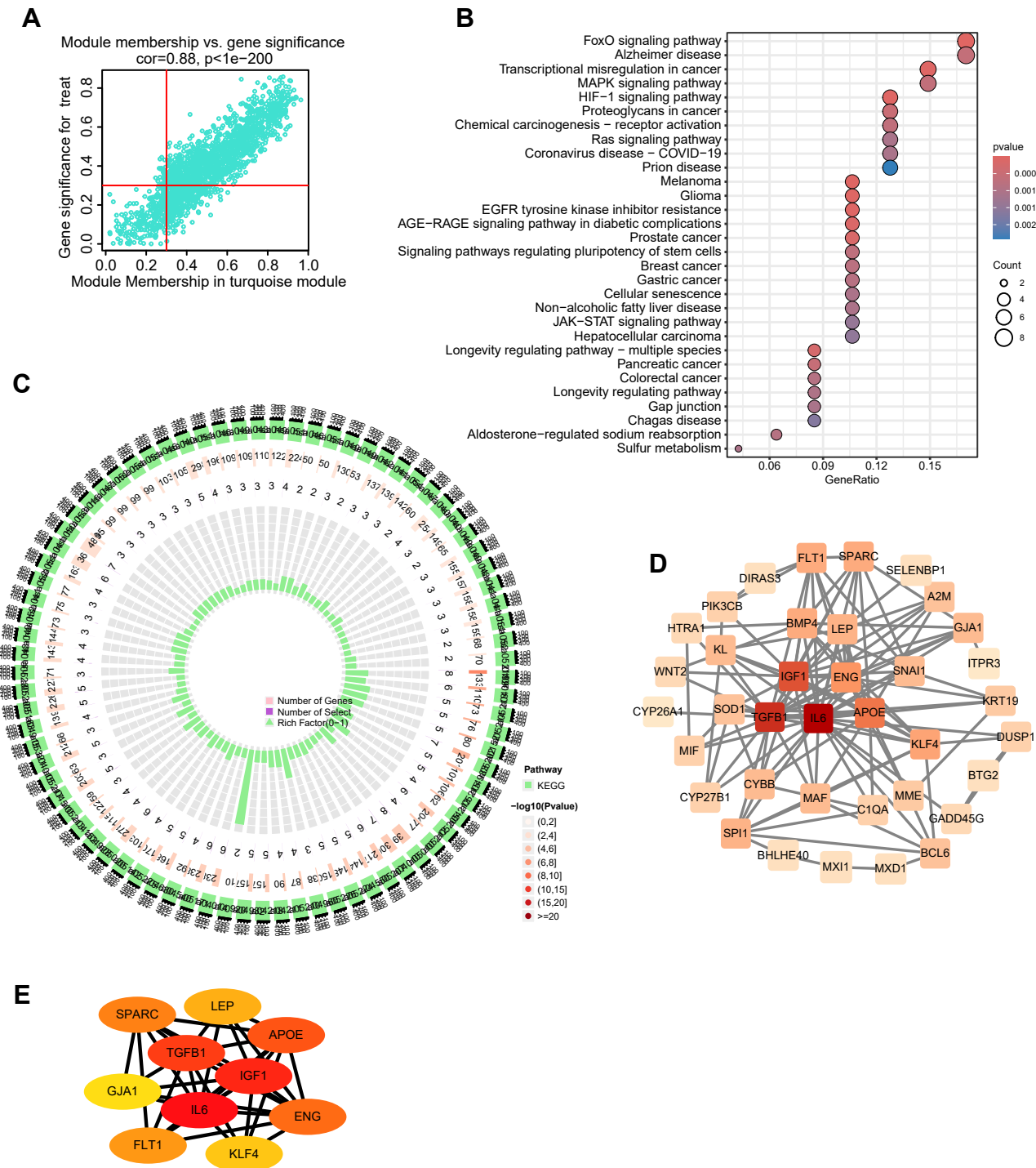

Fig.S3

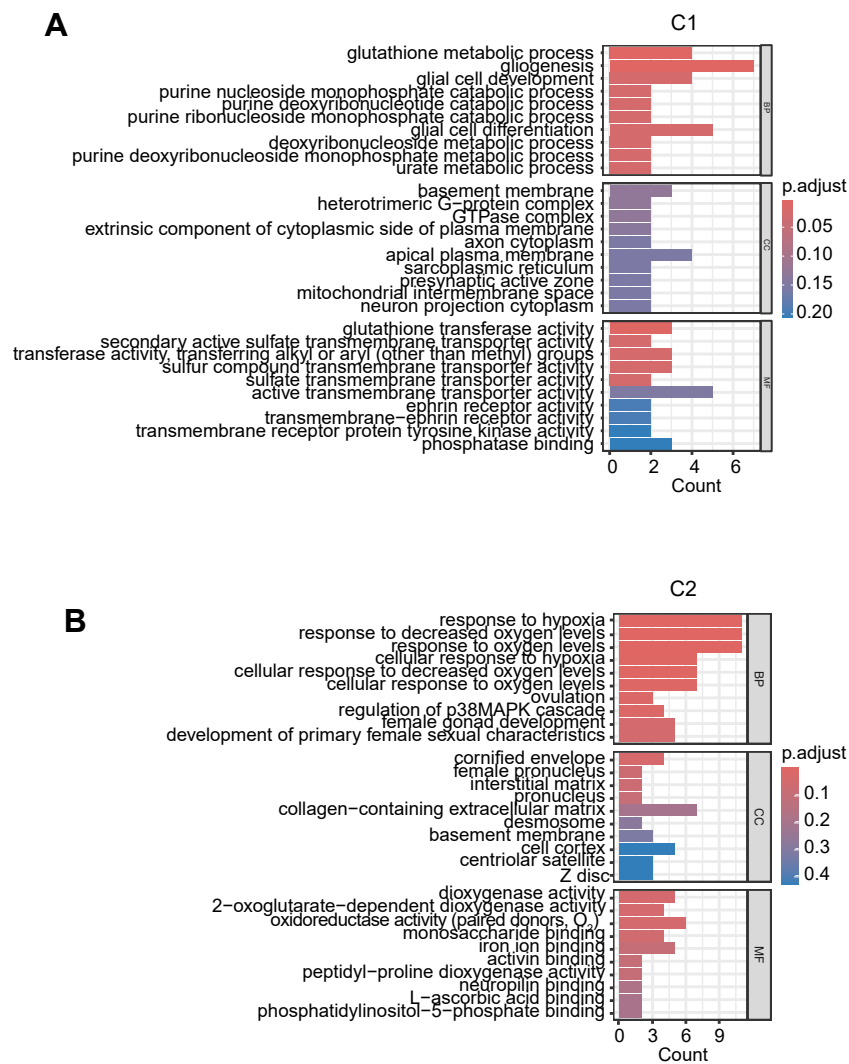

Fig.S4

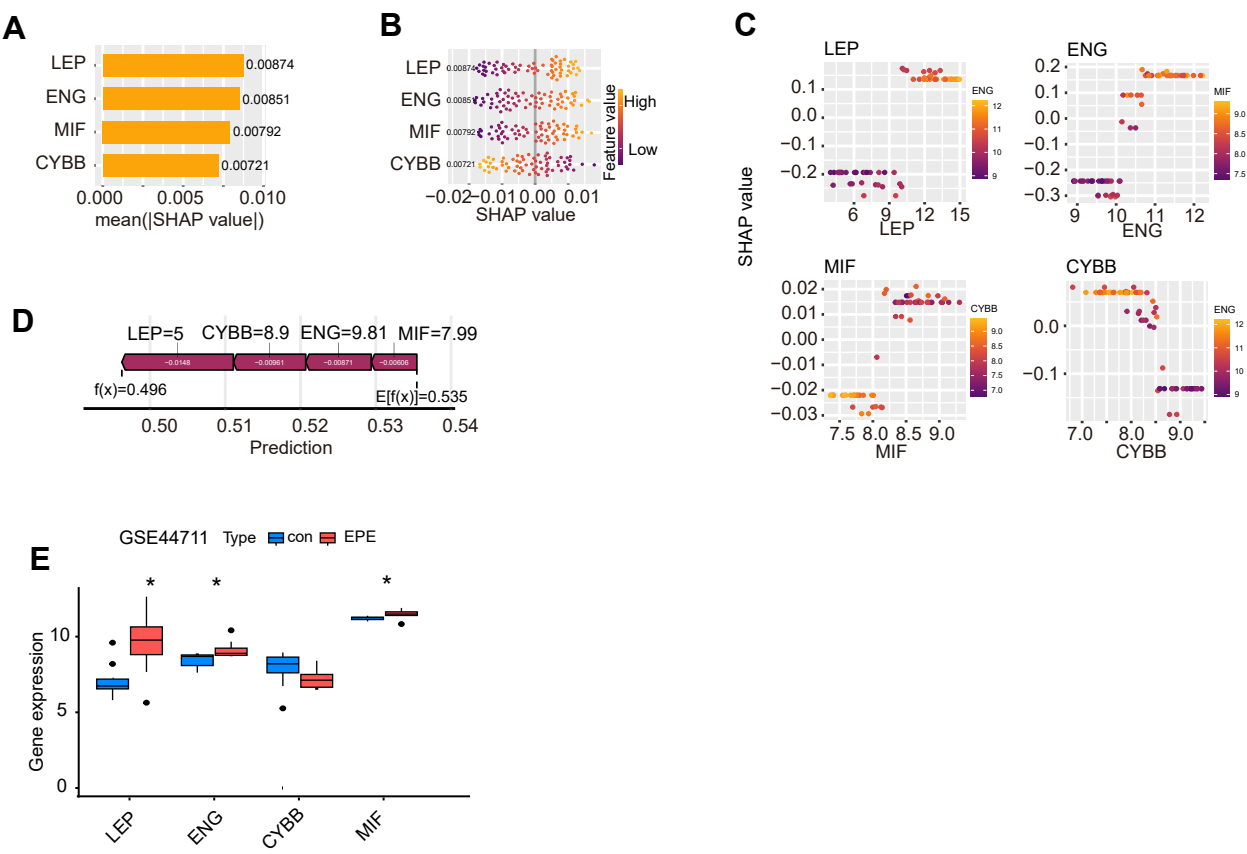

**Fig.S5**

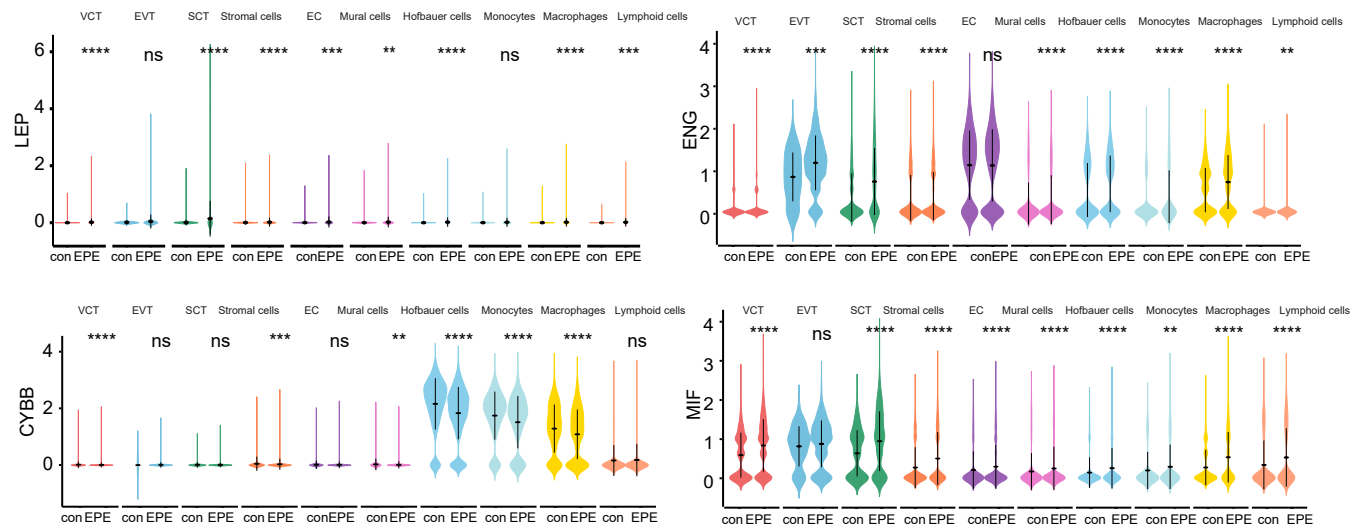

**Fig.S6**

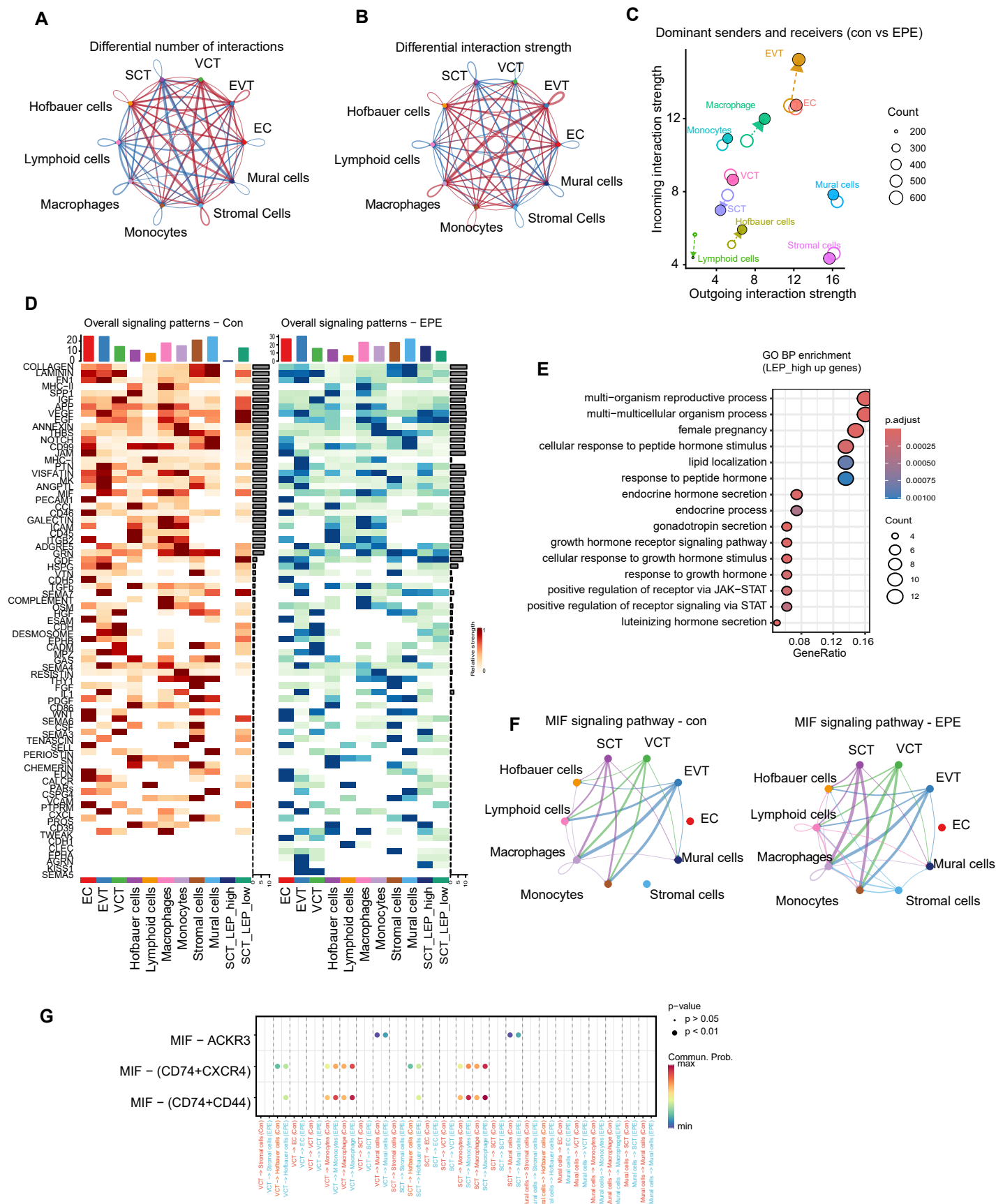

Fig.S7

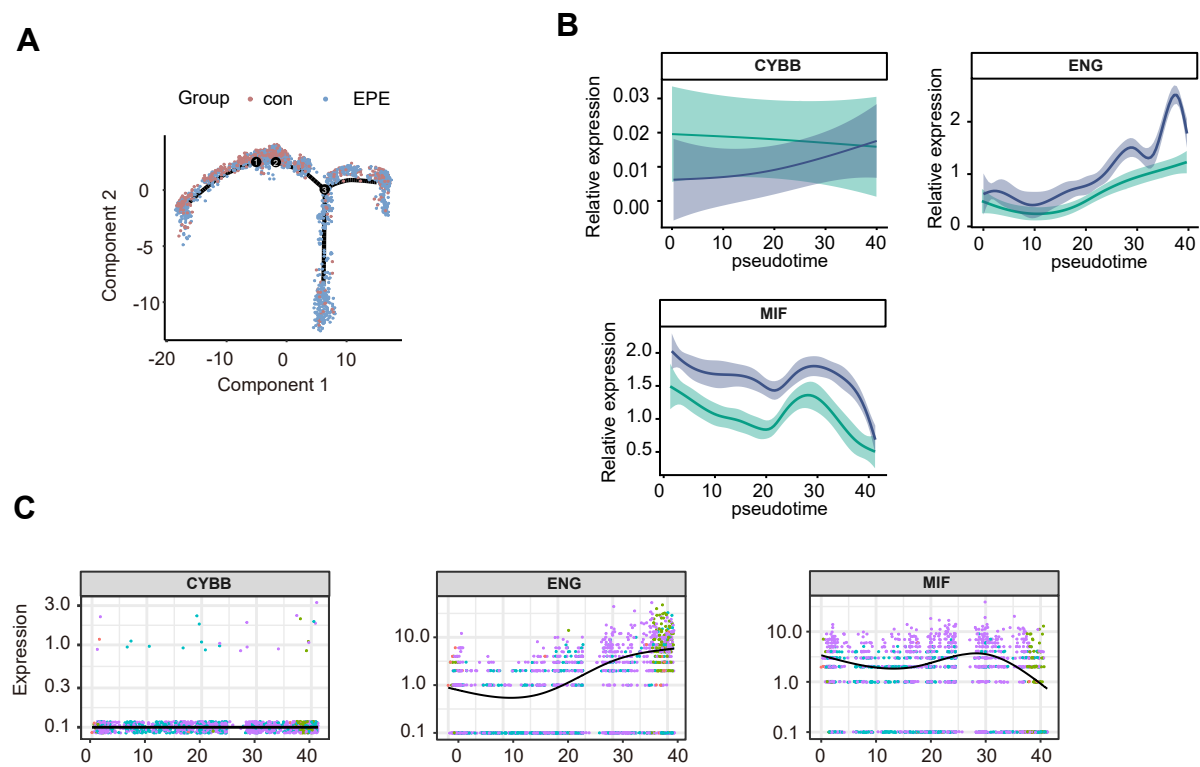

Supplement: Supplementary Figure 1 — Additional preprocessing and enrichment results for the integrated bulk transcriptomic datasets. (a) PCA of the three placental transcriptomic datasets before batch correction. (b) Distribution of normalized expression values across samples after preprocessing. (c) Circular GO enrichment plot of DEGs across BP, CC and MF categories. [file DataSheet1.pdf]
